# Genetically Predicted Expression of Autophagy-Related Genes and Thoracic Aortic Aging: A Mendelian Randomization Study

**DOI:** 10.1161/HYPERTENSIONAHA.125.25181

**Published:** 2025-08-20

**Authors:** Karim Kohansal Vajargah, Marie-Joe Dib, Cameron Beeche, Hamed Tavolinejad, Stephen Burgess, Julio A. Chirinos

**Affiliations:** 1 Prevention of Metabolic Disorders Research Center, Research Institute for Metabolic and Obesity Disorders, Research Institute for Endocrine Sciences, Shahid Beheshti University of Medical Sciences, Tehran, Iran; 2 Sequoia Genetics Ltd, London, United Kingdom; 3 Division of Cardiovascular Medicine, Hospital of the University of Pennsylvania, 3400 Spruce Street, Philadelphia, PA, 19104; 4 Department of Bioengineering, University of Pennsylvania, 3400 Spruce Street, Philadelphia, PA, 19104; 5 University of Pennsylvania Perelman School of Medicine, Philadelphia, PA, 3400 Spruce Street, Philadelphia, PA, 19104; 6 MRC Biostatistics Unit, University of Cambridge, Cambridge CB2 0SR, UK; 7 Department of Public Health and Primary Care, University of Cambridge, Cambridge CB2 0SR, UK

**Keywords:** arterial stiffness, autophagy, aortic aging, early vascular aging

Autophagy is an evolutionarily conserved cellular process essential for maintaining homeostasis and supporting complex physiological functions. As interest in life expectancy and the biology of aging grows, autophagy has emerged as a key direct regulator in the mechanisms underlying both cardiac and vascular aging (1). Large conduit arteries such as the aorta undergo age-related structural remodeling, marked by wall stiffening, luminal dilation, and elongation. Early aortic aging imposes adverse pulsatile hemodynamic patterns on the heart and peripheral organs and contributes to elevated cardiovascular risk (2). While cardiac aging has been extensively studied, aortic aging and its connection to autophagy remain less explored in humans. In this study, we aimed to investigate the putative causal role of proteins related to autophagy on aortic aging using Mendelian randomization (MR) and Bayesian colocalization analyses.

To define our exposures, we selected 270 genes involved in the autophagy pathway based on annotations from KEGG, Reactome, and evidence from the published literature. Genetic instruments for these genes were selected using *cis*-expression quantitative trait loci (*cis*-eQTL) data obtained from two sources: aortic tissue (*n*=472) from the GTEx v10 project and whole blood samples (*n*=31,684) from the eQTLGen consortium. As described in detail previously (3), thoracic aortic aging was assessed using three-dimensional aortic geometry parameters (diameter, length, curvature, and tortuosity, measured across the entire thoracic aorta and its subsegments) extracted from axial steady-state free precession MRI images in 41,926 UK Biobank participants, followed by a genome-wide association study, which was used as the outcome in this MR study. We performed two-sample MR to assess putative causal associations between autophagy-related gene expression and aortic aging. Genetic instruments were selected from *cis*-eQTL datasets using a genome-wide significance threshold (*P*<5×10^−8^), followed by linkage disequilibrium (LD) clumping to ensure independence between variants. The Wald ratio was used for single-SNP instruments, and inverse-variance weighted MR for multiple-SNP instruments. To correct for multiple testing, we applied a 5% false discovery rate (FDR). Statistically significant MR results were further evaluated using Bayesian colocalization as sensitivity, to test for shared causal variants between exposure and outcome.

After harmonizing the data and conducting two-sample MR using *cis*-eQTL genetic instruments for 270 autophagy-related genes, we identified six proteins whose genetically predicted expression was significantly associated with aortic aging in either whole blood or aortic tissue. These results are summarized in Figure 1. Ubiquitin-Conjugating Enzyme E2 L3 (UBE2L3) was found to be significant in both whole blood and aortic tissue. In whole blood, the genetically predicted expression level of UBE2L3 was positively associated with aortic aging (*β*=0.16; 95%CI=0.07, 0.24; *P*_*FDR*_=0.03). Similarly, in aortic tissue, UBE2L3 showed an association with aortic aging (*β*=0.91; 95%CI=0.43, 1.39; *P*_*FDR*_<0.001). For Tubulin Alpha 1c (TUBA1C), the genetically predicted inhibition of expression was associated with aortic aging in whole blood (*β*=-0.22; 95%CI=-0.35, -0.09; *P*_*FDR*_=0.042), but no significant association was found in aortic tissue. Similarly, for GABA Type A Receptor Associated Protein Like-1 (GABARAPL1), we observed no significant association for eQTLs derived from aortic tissue, but in whole blood, inhibition of its expression was significantly associated with aortic aging (*β*=-0.34; 95%CI=-0.54, -0.14; *P*_*FDR*_=0.042). Autophagy-Related 13 (ATG13), another autophagy-related molecule, showed significant associations with aortic aging in both tissues. Genetically predicted inhibition of ATG13 expression was associated with aortic aging using whole-blood eQTLs (*β*=-0.95; 95%CI=-1.48, -0.42; *P*_*FDR*_=0.031) and aortic tissue eQTLs (*β*=-0.57; 95%CI=-0.86, -0.27; *P*_*FDR*_<0.001). The genetically predicted expression level of Microtubule Associated Protein 1 Light Chain 3 Alpha (MAP1LC3A) showed a significant association only in aortic tissue (*β*=-0.26; 95%CI=-0.42, -0.11; *P*_*FDR*_=0.034). Finally, Mitogen-Activated Protein Kinase Kinase 2 (MAP2K2) was positively associated with aortic aging in aortic tissue (*β*=0.69; 95%CI=0.36, 1.03; *P*_*FDR*_<0.001), but no significant association was found for whole blood. Bayesian colocalization analysis further confirmed the findings, with evidence of colocalization (posterior probability of H4=83.8%) for UBE2L3, suggesting shared causal variants between UBE2L3 expression and aortic aging.

We identified six autophagy-related genes whose genetically predicted expression levels exhibited putative causal associations with thoracic aortic aging. There was robust genetic evidence supporting consistent effects for UBE2L3 across whole blood and aortic artery tissues in MR results, further supported by colocalization indicating a shared causal variant. UBE2L3, a ubiquitin conjugating enzyme, plays a key role in regulating inflammation by targeting pro-IL-1β for proteasomal degradation, thereby limiting the production of excess mature IL-1β (4). Given that chronic low-grade inflammation is a hallmark of aging, impaired UBE2L3 function could lead to heightened inflammatory signaling and contribute to aortic aging. Importantly, UBE2L3 itself is degraded via the proteasome rather than autophagy, suggesting a complex interplay between proteostasis and inflammation in the aging vasculature (5). These findings highlight UBE2L3 as a potential molecular target for mitigating age-related vascular decline, pending future experimental validation. Further studies in diverse populations and ancestries beyond the general European population analyzed here are also required.

## Figures and Tables

**Figure 1:**
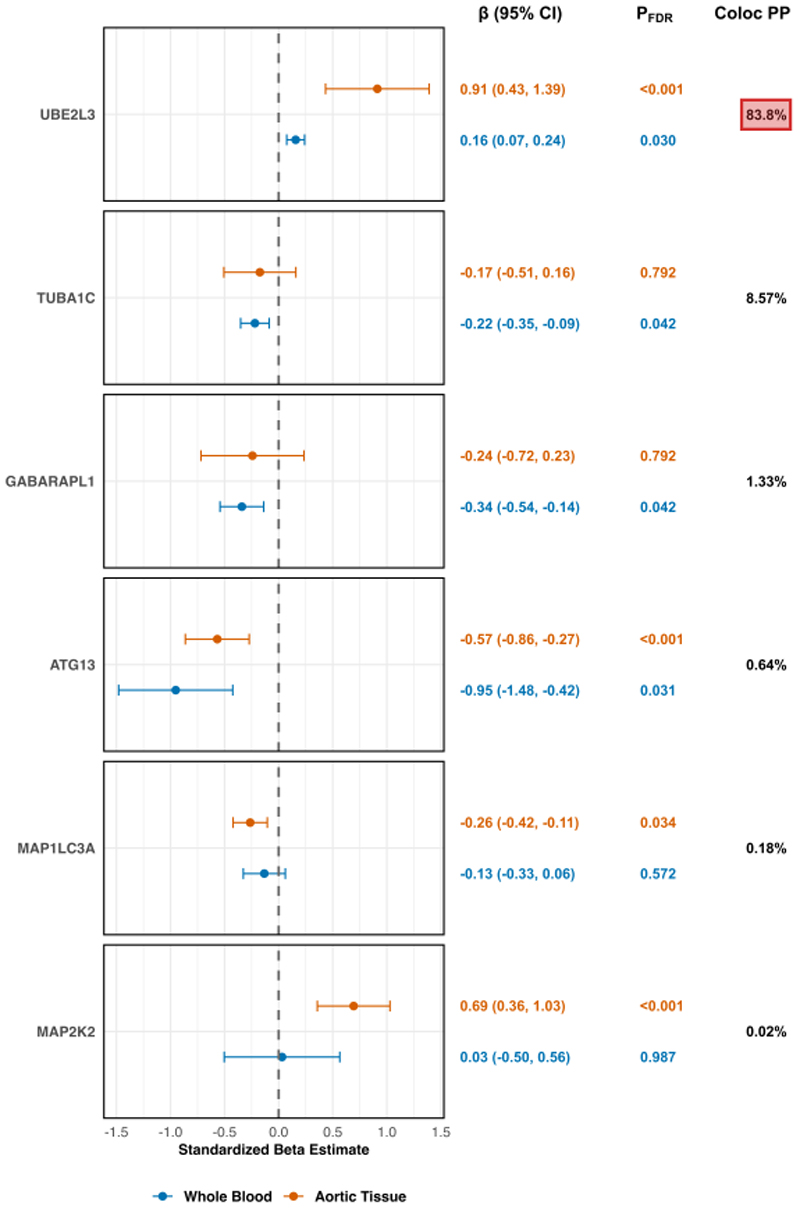
The association between genetically predicted expression levels of autophagy-related genes and thoracic aortic aging (both scaled per 1 standard deviation change) in two-sample Mendelian Randomization analyses. The plot also presents the corresponding Bayesian colocalization posterior probabilities, indicating the likelihood of shared causal variants between genetically predicted gene expression levels and aortic aging. Gene expression data were derived from whole blood (eQTLGen, *n*=31,684) and aortic tissue (GTEx v10, *n*=472). UBE2L3: Ubiquitin-Conjugating Enzyme E2 L3; TUBA1C: Tubulin Alpha 1c; GABARAPL1: GABA Type A Receptor Associated Protein Like 1; ATG13: Autophagy-Related 13; MAP1LC3A: Microtubule Associated Protein 1 Light Chain 3 Alpha; MAP2K2: Mitogen-Activated Protein Kinase Kinase 2; FDR: False Discovery Rate; Coloc PP: Colocalization Posterior Probability
